# The emergence of land systems as the nexus for sustainability transformations: *This article belongs to *Ambio*'s 50th Anniversary Collection. Theme: Agriculture land use*

**DOI:** 10.1007/s13280-021-01519-9

**Published:** 2021-03-13

**Authors:** Ariane de Bremond

**Affiliations:** Centre for Development and Environment (CDE), University of Bern, Mittelstrasse 43, 3012 Bern, Switzerland

**Keywords:** Global change science, Land-cover land-use change, Land system science, Land use, Socio-ecological system (SES), Sustainability transformations

## Abstract

This perspective recognizes the seminal *Ambio* articles of Sombroek et al. (1993), Turner et al. (1994) and Brussaard et al. (1997), identifying their individual and collective role in laying the ground work for a global change research agenda on land and its human use through increased understanding of terrestrial ecosystem dynamics and global change, and furthering nascent interdisciplinary efforts within the global change science community to better understand the ‘human driving forces’ of change. From these efforts, land system science, as a systemic science focused on complex socio-ecological interactions around land use and associated trade-offs and synergies, emerges as an ‘interdiscipline’ challenged to better understand land systems as the ‘meeting ground’ for multiple claims on land for biodiversity, carbon, livelihoods, food production among others, and support pathways to sustainability for people and nature.

## Introduction

Land lies at the nexus of crucial societal and environmental challenges, offering a means to address food security and livelihoods, ending poverty, women’s empowerment, access to water, biodiversity loss, and climate change among others. Land provides the bridge between the UN’s Sustainable Development Goals, as decisions on land use—governed by social interactions among diverse stakeholders and institutions—serve as the very pathways through which humans’ and nature’s well-being can be secured. A key process of global environmental change is change in land use: the purposes and activities through which people interact with land and terrestrial ecosystems, which at the same time, generates many sustainability challenges (Meyfroidt et al. 2018). More than three quarters of the Earth’s terrestrial surface is currently managed to meet a combination of human needs via agriculture, forestry, and settlements, (Ellis 2015, Ramankutty et al. 2018) and producers support consumers from progressively distant places across the world. In addition to rising demands on agricultural production to support more people with changing diets, landscapes play an increasingly important role in sustaining a wider variety of services such as flood control, water purification, and cultural and esthetic values; in securing global commons by sequestration of carbon emissions in vegetation and soils; and in protection of biodiversity (Díaz et al. 2019). More and more, land is a limited resource with multiple, growing, and competing claims being placed on it by new and old actors alike.

The scientific community’s contemporary understanding of land as a socioecological system has its roots in the formal, 1990 launch of the International Geosphere Biosphere Programme, whose mission was “to describe and understand the interactive physical, chemical, and biological processes that regulate the total Earth system, the unique environment that it provides for life, the changes that are occurring to this system, and the manner in which they are influenced by human actions” (IGBP 1988). With the recognition that human land use was influencing the chemistry of the air, the diversity of plant and animal species, and the balance of global ecosystems, the concept of global change acquired a new meaning. Scientific disciplines and programmes that in the past operated alone now collaborated to advance the understanding of how humans, as a whole, influence the earth, with specific research areas identified and broken down into ‘core projects,’ the foundation and early growth of an international and interdisciplinary scientific community that continues its evolution today.

Nearly 30 years ago, the pages of this journal offered some of the first glimpses of how scientists in this new and rapidly consolidating global change science community would lay out, craft, and envision core elements of their scientific agenda, new data needs, methodological frontiers and challenges, and importantly, how they would articulate frameworks for future research that would serve to structure and enable international collaboration for a new generation of global change science.

This reflection recognizes three such seminal articles, identifying their individual and collective role in laying the ground work for a global change research agenda on land and its human use. First, the contributions by Sombroek et al. (1993), Turner et al. (1994) and Brussaard (1997) are considered, particularly how they served to advance initial frameworks, methods, and ‘standards,’ building an understanding of terrestrial ecosystem dynamics and global change, and furthering nascent interdisciplinary efforts within the global change science community to better understand the ‘human driving forces’ of change. Next, the emergence and consolidation of land system science (LSS) as an ‘interdiscipline’ (Young and Lutters 2015) and (Turner et al. this issue), and of the Global Land Programme as referent for the LSS community, which have been the succeeding programmatic efforts to build and sustain a community of scientists working together to understand the changing interactions among human systems, the terrestrial biosphere, atmosphere, and other Earth systems, as mediated by human use of land. And finally, the consideration of the articles’ legacy as carried forth through ongoing efforts in land system science, to better understand land systems as the ‘meeting ground’ for multiple claims on land (for biodiversity, carbon sequestration or food security among others), and asking, how is land system science poised to contribute to devising pathways to sustainability and to global environmental and human development policy priorities?

## Towards an understanding of terrestrial ecosystem dynamics and global change

Each of the articles in the *Ambio* anniversary collection on agriculture and land use changes employed the emerging systems approach to studying earth at the time. For Sombroek et al. (1993) the refinement of estimates of global soil carbon pools using new digitized maps generated through international scientific collaboration furthered understandings of the importance of sound organic matter management and set the stage for further exploration of soil-carbon sequestering. In so doing, Sombroek’s analysis supported an objective of the Global Change and Terrestrial ecosystems (GCTE) project of IGBP, launched in 1992, to predict the effects of changes on climate, atmospheric composition, and land use on terrestrial ecosystems, including agriculture, forestry and soils, and to consider the terrestrial carbon cycle with an emphasis on underlying drivers and processes of contemporary and future carbon quantities (fluxes and pools) (Walker and Steffen 1996), thus setting the stage for decades of research on the critical role of soils in climate change mitigation and adaptation.

Brussard (1997) similarly structures his review of biodiversity and ecosystem functioning in soil as related to human land use, specifically, the need to address the loss of functioning in soils associated with intensive agriculture, forest disturbance, pollution and global environmental change more broadly. Again, new research agendas are set around experimental approaches involving long-term and large-scale experimentation approaches—those requiring broad, coordinated, international collaboration— coupled with then cutting-edge methods of geostatistics and Geographic Information Systems.

The path forward for the integrated study of dynamic interactions between human and environmental systems through land use, land cover and land management changes was most centrally charted by the 1994 article by Turner et al. in outlining the research plan for a Land Use Cover Change (LUCC) Project, one of the first joint projects of IGBP and newer Human Dimensions of Global Environmental Change Program (HDP). In the pursuit of understanding land-use land cover change in relationship to global environmental change, the ‘cause to cover’ relationship between physical land transformation and its social drivers, though not a new concern, become firmly squared on building understanding of the deeply social human driving forces of land use change. It set out an interdisciplinary agenda that identified large scale ‘cause to cover relationships’ and a common protocol for case studies by which the cause-to-cover dynamics across contexts could be specified, which could be fed into the development of global land use and land cover models. In this way, the program outlined in the article set a broad, challenging, and ambitious agenda for research on land systems that the now growing community of land system scientists continues to progress.

## Advancing the science of land systems through the Global Land Programme

The rich reflection of Turner et al. (this issue) on Turner et al. (1994) charts the emergence of land system science and its contemporary referent community—the Global Land Programme, over the last decades, tracing key advances in the field including improved identification of the causes of land change; major progress in remote sensing and ‘big data’ applications and in modelling of land systems; deeper integration of social and environmental subsystem dynamics; and not least, in deepening understanding of land systems as solutions to the global change and sustainability challenges of our time.

By the late 2000s, land science had produced a wealth of methodological innovations and empirical observations on land-cover and land-use change, from patterns and processes to causes, but it had also reached a critical juncture (Rindfuss et al. 2008): the need for comparison and generalization (the ability to move beyond a specific case study or a particular model) and further theory-building that could permit LSS to navigate between context dependence and very high level generalization. More recently, LSS is attending to theory-building through efforts to map middle-range theories (e.g., land-use expansion and intensification, small holder subsistence land use, induced intensification and land rent, among others) (Meyfroidt et al. 2018), and the provision a framework for an eventual inclusive theory of land use or sets of theories (Turner et al. 2020a, b). Importantly, this key challenge to the scientific enterprise is also central to the ability to provide insights and evidence to better inform specific policy responses (Turner et al. 2007) and to produce knowledge on systemic entry points for global transformations to sustainable development (Messerli et al. 2019).

As land science has grown to become an ‘interdiscipline’ it has sustained progressions in knowledge. Researchers spanning the natural, physical, and social sciences are working to address critical knowledge gaps in our understanding of land system change with respect to human behaviors (cognitions, culture, and decision-making) for example; also, to better incorporate feedbacks between environmental change and human activities; land use-intensity and management; and globalization, trade-flows, and distant causes as exemplified by a growing field of supply chain governance studies.

Over this same period, the ‘umbrella’ programmes of the global environmental change community have undergone their own transition, amidst growing calls for integrated social-environmental and/or global environmental change science (GEC) to better mobilize knowledge for the pursuit of sustainable development. Most recently, the Future Earth research initiative—of which the Global Land Programme (GLP) is a ‘global research project’ (GRP) representing the land systems science (LSS) community—aims for its collection of programs and networks^1^ to “enable a systems-based approach to global sustainability through the Sustainable Development Goals” and “provide transdisciplinary science needed to make them actionable” (Future Earth 2018). Land system science, as a systemic science focused on complex socio-ecological interactions around land use and associated trade-offs and synergies and transdisciplinary approaches, is well placed to contribute to this agenda.

**Fig. 1:**
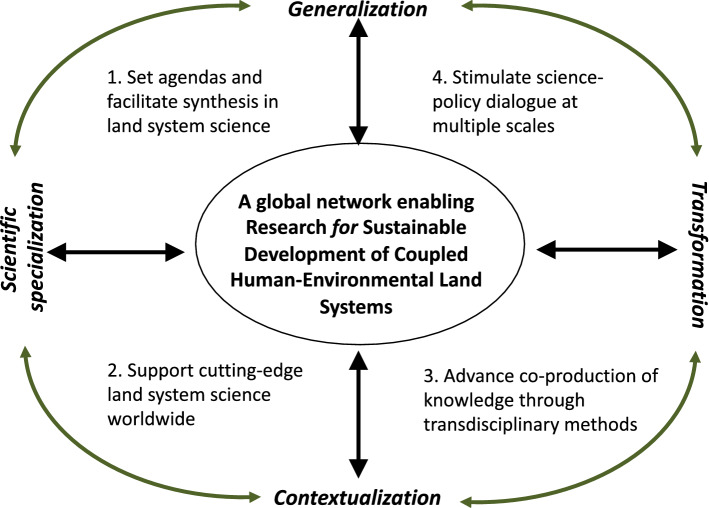
GLP’s mission, overall goals, and objectives 1–4. Adapted from: Hurni and Wiesmann (2014)

Today, GLP’s mission is to enable research for sustainable development of coupled human–environmental land systems (Fig. 1). This mission entails a bridging of scientific innovation (specialization) with societal relevance (transformation) and employs place-based research (contextualization) to feed synthesis-understandings of the patterns and processes of global change (generalization). Accordingly, GLP’s three overall goals are to: (1) Set and drive an agenda for the land systems science community that links scientific advancement to societal relevance; (2) develop new synthesis methods and products to connect contextual understandings to regional and global trends, drivers, and consequences; and (3) support and maintain a vibrant network of scientists enabling that work together with new assemblages of actors including civil society, government, and private sector, in new modes of science–society interaction, towards sustainable development of land systems.

Underlying these goals is the hypothesis that the development of transformative social pathways towards land-related Sustainable Development Goals (SDGs) remains inhibited by fragmented activities of involved stakeholders and their knowledge. While private actors, development practitioners, and policy makers may be strengthening their collaborations, the majority of researchers are still absent from such partnerships.

## Challenges on the horizon

In the 2019 4th Open Science Meeting (OSM) of the Global Land Programme, Scientific Steering Committee member and OSM Science Chair Patrick Meyfroidt reflected on the hybridity of land system science: being both a field of professional study as well as a community of people who have been traveling, more or less together, on a journey. To reflect back on this journey is to see how each of these three *Ambio* articles— now decades old— laid the first guideposts on a map that is still being drawn.

A starting point for the next leg of this ‘journey’ is the recognition that land is a meeting ground, a boundary object, a nexus to which multiple issues, functions, uses, values, and goals for land interact. With multiple claims on land, for biodiversity, carbon, livelihoods, food production, it is essential to understand how these different stakes come together in land, and how complex trade-offs and synergies across these multiple functions and across different scales can be balanced, and solutions navigated and negotiated amongst multiple paradigms, perspectives, and social groups.

For the Sustainable Development Goals (SDGs) and Agenda 2030 to be realized, we must work, first, to harness the great advances in understanding human–environment systems not to identify systemic challenges but rather opportunities for change. And, second, on that basis, to craft concrete pathways through reflexive, engaged approaches with societal actors, increasingly mobilizing the urgently needed scientific knowledge about social, behavioral, economic, and technological levers for change. This is where the growing community of land systems science now finds itself today: working to understanding land as a cause and consequence of global change and as the nexus for sustainability transformations.

## Footnotes

1 The Global Research Projects (GRPs), Knowledge Action Networks, (KANS), and Future Earth National Committees are considered to be main vehicles for the activities of Future Earth.
